# Multi-parametric magnetic resonance imaging of liver regeneration in a standardized partial hepatectomy rat model

**DOI:** 10.1186/s12876-022-02517-1

**Published:** 2022-10-09

**Authors:** Caixin Qiu, Shuangshuang Xie, Yajie Sun, Yongquan Yu, Kun Zhang, Xuyang Wang, Jinxia Zhu, Robert Grimm, Wen Shen

**Affiliations:** 1 Department of Radiology, Tianjin First Central Hospital, Tianjin Institute of Imaging Medicine, No.24 Fukang Road, Nankai District, Tianjin, 300192 China; 2 Department of Radiology, Weihai Central Hospital, Shandong, China; 3 Medical College of Nankai University, Tianjin, China; 4 MR Collaboration, Siemens Healthcare Ltd., Beijing, China; 5 MR Application Predevelopment, Siemens Healthcare GmbH, Erlangen, Germany

**Keywords:** Magnetic resonance imaging (MRI), Liver regeneration, Hepatectomy, Diffusion kurtosis imaging (DKI)

## Abstract

**Background:**

We aimed to evaluate the correlation between the pathological changes and multi-parameter MRI characteristics of liver regeneration (LR) in a standard partial hepatectomy (PH) rat model.

**Methods:**

Seventy Sprague–Dawley rats were randomly divided into two groups: MR scan group (n = 14) and pathologic analysis (PA) group (n = 56). All 14 rats in the MR group underwent liver T1 mapping, T2 mapping, and diffusion kurtosis imaging before and the 1st, 2nd, 3rd, 5th, 7th, 14th, and 21st day after 70% hepatectomy. Seven rats in the PA group were euthanized at each time point to determine Ki-67 indices, hepatocyte size (HTS), steatosis grade, and inflammation score.

**Results:**

Liver T1 and T2 values increased to maximum on day 2 (*P* < 0.001 vs. baseline), D and K values decreased to minimum on day 3 and 2, respectively (*P* < 0.001 vs. baseline), then all parameters returned to baseline gradually. Hepatocyte Ki-67, hepatocyte size, steatosis grade, and inflammation score initially increased after surgery (*P* < 0.05 vs. baseline), followed by a gradual decline over time. Both T2 and K values correlated well with Ki-67 indices (r = 0.765 and − 0.807, respectively; both *P* < 0.001), inflammation (r = 0.809 and − 0.724, respectively; both *P* < 0.001), steatosis grade (r = 0.814 and − 0.725, respectively; both *P* < 0.001), and HTS (r = 0.830 and − 0.615, respectively; both *P* < 0.001).

**Conclusions:**

PH induced liver changes that can be observed on MRI. The MRI parameters correlate with the LR activity and allow monitoring of LR process.

## Background

Partial hepatectomy (PH) is a surgical treatment for liver tumors and a necessary surgical procedure for living donor liver transplantation [1]. Residual liver regeneration is closely related to the prognosis of liver tumor patients, donors, and recipients. Although PH has been widely used in clinical practice and is a relatively safe procedure, patients still face the risk of surgical failure due to liver regeneration failure [2]. Therefore, finding an appropriate method to monitor liver regeneration is of great concern to hepatobiliary surgeons. At present, computed tomography or magnetic resonance imaging (MRI) measurement of liver volume to monitor liver regeneration is a commonly used method in clinical practice [3–6]. However, volume recovery may be overestimated and cannot reflect the microscopic changes of liver parenchyma [6]. Previous studies have reported that hypertrophy and hepatocyte division contribute to liver regeneration [7]. In this process, the number of residual hepatocytes, hepatocyte size, tissue structure, and material metabolism changed significantly [8]. MRI offers a non-invasive option for assessing biomarkers associated with pathophysiologic changes. MR T1 mapping and T2 mapping scanning can assess water molecule content, collagen fiber deposition, inflammation, and lipid infiltration of the tissue, corresponding to possible changes during liver regeneration [9, 10]. Diffusion kurtosis imaging (DKI) is sensitive to tissue microstructure and conducive to reflecting the actual situation in tissues, providing an opportunity to gain insights into the state of liver diffusivities and tissue microstructural complexity [11–14]. Published studies have investigated the association between a single MRI feature and liver regeneration, but few have comprehensively assessed liver regeneration and their associations pathologically and radiologically [15–17].

In this study, we used multi-parametric MRI to longitudinally observe liver changes in a standardized rat model after 70% PH. Our purpose was to determine whether multi-parametric MRI values from T1 and T2 mapping and DKI could be used to correlate microscopic liver regeneration changes to residual liver regeneration indices, and thus to lay an experimental basis for clinicians to find a non-invasive method for assessing liver regeneration.

## Materials and methods

### Animals

**Fig. 1:**
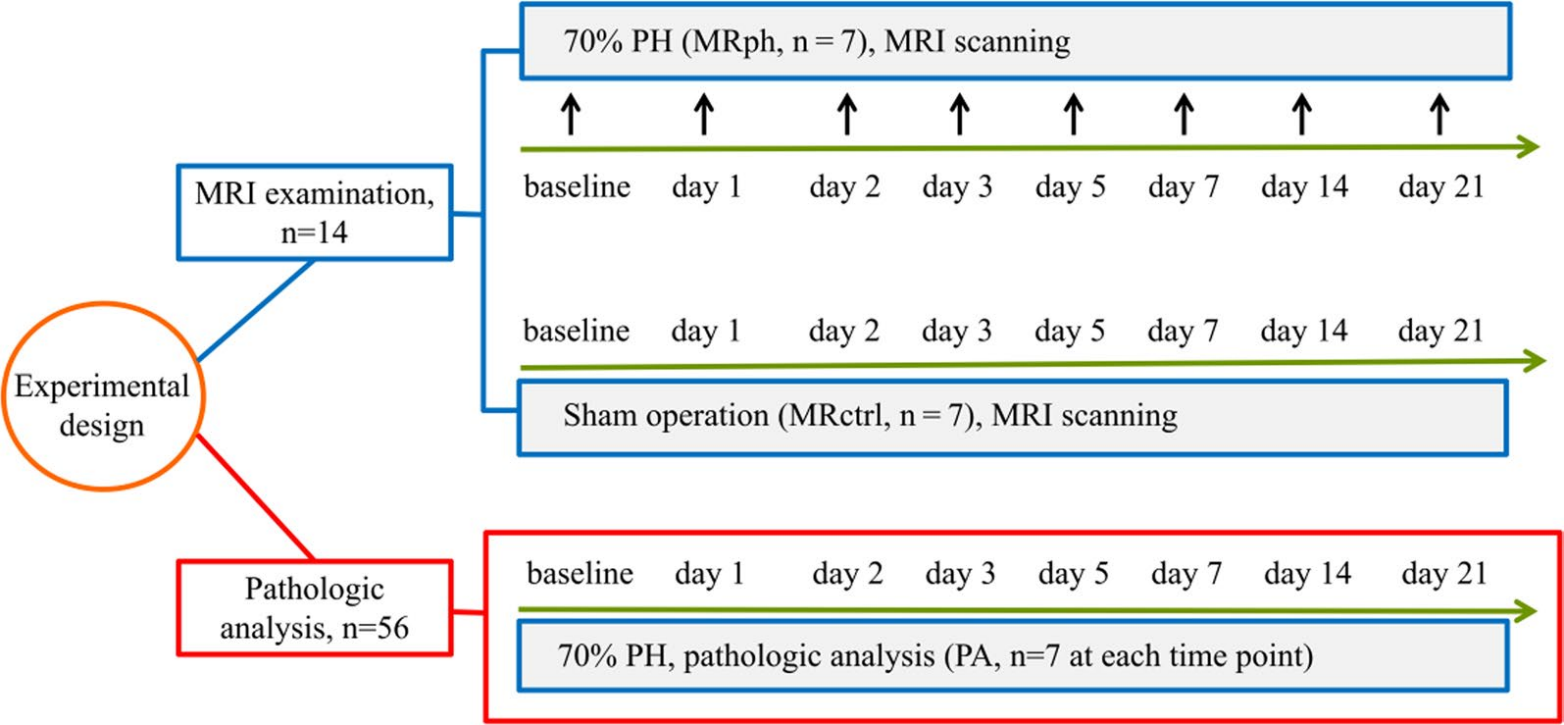
The experimental protocol in this study. PH = partial hepatectomy

A total of 70 male Sprague–Dawley rats aged 7 to 8 weeks and weighing 260 ± 30 g were obtained (China Food and Drug Control Research Institute, Beijing, China). Animals were housed in the animal facility of the Key Laboratory of Organ Transplantation of Tianjin with unrestricted access to standard chow and water. All experiments were approved by the NANKAI University Animal Experimentation Ethics Committee and performed according to the University's animal care guidelines. Rats were randomly divided into two groups: an MR scan (MR) group (n = 14) and a pathologic analysis (PA) group (n = 56). The MR group was further divided into a 70% PH subgroup (MRph, n = 7) and a control subgroup (MRctrl, n = 7). Before surgery, all 14 rats in the MR group underwent MR imaging, and a random 7 of the 56 rats in the PA group were euthanized for pathologic analysis to establish baseline values. The 7 rats in the MRph subgroup and the 56 rats in the PA group underwent a 70% PH, and the 7 rats in the MRcrtl subgroup underwent a sham operation. MR scan group underwent liver T1 mapping, T2 mapping, and DKI examinations before and on the 1st, 2nd, 3rd, 5th, 7th, 14th, and the 21st day after surgery. The PA group, 56 rats in total, randomly selected seven with 70% PH at each corresponding time point above for pathologic analysis, including Ki-67 indices, hepatocyte sise (area), inflammation score, and steatosis grade (Fig. 1).

### Anesthesia and surgical procedures

The surgical procedures were performed under inhalation anesthesia. Induction was performed in a glass box with a mixture of 2% isoflurane in oxygen (1.0 L/min) (Ruiwode; Shenzhen, China). During surgery, anesthesia was maintained with 2% isoflurane in oxygen through a mask covering the animal's mouth and nose. The animal placed supine on an operating plate underwent a midline laparotomy, and the liver was freed from its ligaments. For the MRph and PA groups, the common pedicle of the left lateral and median lobes was ligated with a 4–0 suture, and the lobes were resected. The abdomen was then closed with a 3–0 suture. For the MRcrtl group, the left lateral and medial lobes were freed from ligaments. The abdomen was then closed with a 3–0 suture.

### Magnetic resonance imaging

All the rats underwent MRI examinations on a 3 T MR scanner (MAGNETOM Prisma, Siemens Healthcare, Erlangen, Germany) with an 8-channel animal coil (Chenguang, Shanghai, China). The rats were anesthetized with isoflurane inhalation and placed in a prone position to reduce respiratory motion. T1 weighted imaging (T1WI) was performed for liver segmentation. A B1 mapping using turbo-FLASH sequence was acquired for T1 map correction. The detailed parameters were: repetition time (TR) = 5050 ms, echo time (TE) = 1.83 ms, Flip angle = 8°, field of view (FOV) = 360 × 293 mm^2^, slice thickness = 8 mm, matrix = 64 × 52, voxel size = 5.6 × 5.6 × 8 mm^3^, acquisition time = 10 s. The T1 mapping was acquired using a 3D gradient echo sequence with the volumetric interpolated breath-hold examination (VIBE) sequence with a dual-flip-angle method. Sequence parameters were: TR = 6.30 ms, TE = 2.88 ms, Flip angles = 3° and 15°, FOV = 120 × 98 mm^2^, slice thickness = 3 mm, matrix = 192 × 156, voxel size = 0.6 × 0.6 × 3 mm^3^, bandwidth = 300 Hz/px, acquisition time = 2 min 27 s. T2 mapping was acquired using a multi-echo spin echo sequence, with following parameters: TR = 2000 ms, 6 TEs = 13/26/39/52/65/78 ms, FOV = 120 × 120 mm^2^, slice thickness = 3 mm, matrix = 128 × 96, reconstructed voxel size = 0.5 × 0.5 × 3 mm^3^, bandwidth = 201 Hz/px, acquisition time = 4 min 50 s. DKI was performed using a free-breathing single-shot EPI sequence with the following parameters: TR = 2300 ms, TE = 74 ms, FOV = 120 × 98 mm^2^, slice thickness = 3 mm, matrix = 128 × 98, reconstructed voxel size = 0.5 × 0.5 × 3 mm^3^, acquisition time = 4 min 43 s. Five b-values (0, 500, 1000, 1500, and 2000 s/mm^2^) were applied in 3 directions.

### MRI liver volume measurement

Regions of interest (ROI) were manually drawn using T1WI by two independent blinded examiners. The liver volume was measured using software from an IQQA-3D-Liver workstation (EDDA Technologies, USA), allowing quantitative ROI analysis. Liver volume was determined by summing the manual segmentation from each slice.

### Imaging data analysis

Parametric T1 and T2 maps were generated inline after data acquisition using the MapIt processing tool (Siemens Healthcare, Erlangen, Germany). B1 field correction was automatically executed during the calculation of the T1 map. DKI data were post-processed using a prototype software (MR Body Diffusion Toolbox, Siemens Healthcare, Erlangen, Germany), the DKI-derived parameters (D and K values) were obtained. According to the original T1 and T2 images, avoiding large vessels, artifacts, and the liver border area, five ROIs were placed on T1 and T2 maps and the average of the T1 and T2 values is calculated. By referring to the same position of T1 and T2 maps, the ROIs were placed on the ADC map to automatically obtain the D and K values at the same time. The average of the five values was used as the final ROI. Two experienced radiologists analyzed all images in a blinded manner.

### Histology and immunohistochemistry

Liver tissue was fixed in a buffered 4% formaldehyde solution, embedded in paraffin, stained with hematoxylin–eosin (HE) and Sirius red using standard histologic techniques. Liver samples were stained with HE to observe the hepatic pathologic structure and determine the hepatocyte size, steatosis grade, and inflammation score. All pathologic specimens were reviewed by an experienced pathologist. Hepatocyte size (area) was determined by measuring ten hepatocytes in high-power fields (× 400) [7]. The grade of steatosis is based on the proportion of hepatocytes containing visible lipid and is expressed semi-quantitatively on a scale of 0–3 (S0, < 5%; S1, 5–33%; S2, 33–66%; S3, > 66%) [18]. The activity of inflammation was assessed using modified Ishak score [scale 0–8 (steatosis 0–3, lobular inflammation 0–3, and hepatocyte ballooning 0–2)] [19]. Immunohistochemical staining was performed to determine the proliferative index Ki-67 (1:100; Abcam, Cambridge, UK). The number and ratio of Ki-67-positive cells were determined by manual counting in five high-power random fields (× 400) per slide.

### Statistics

Statistical analysis was performed on SPSS Version 22 for Mac (SPSS 22; IBM Company, Chicago, IL). The normal distribution of MR parameters in the MRph and MRctrl groups was tested using the Shapiro–Wilk test. MR parameters were compared by using repeated-measures one-way analysis of variance. Post-hoc pair-wise comparisons with Bonferroni corrections were performed to compare the parameters at different time points. The liver volume and pathologic indices at different time points were compared by one-way ANOVAs and the Bonferroni post hoc test. The correlations between MR and liver regeneration parameters were determined using Pearson correlation coefficient analysis or Spearman's rank correlation analysis. The threshold of significance was set to 0.05.

## Results

### MR parameters analysis

**Fig. 2:**
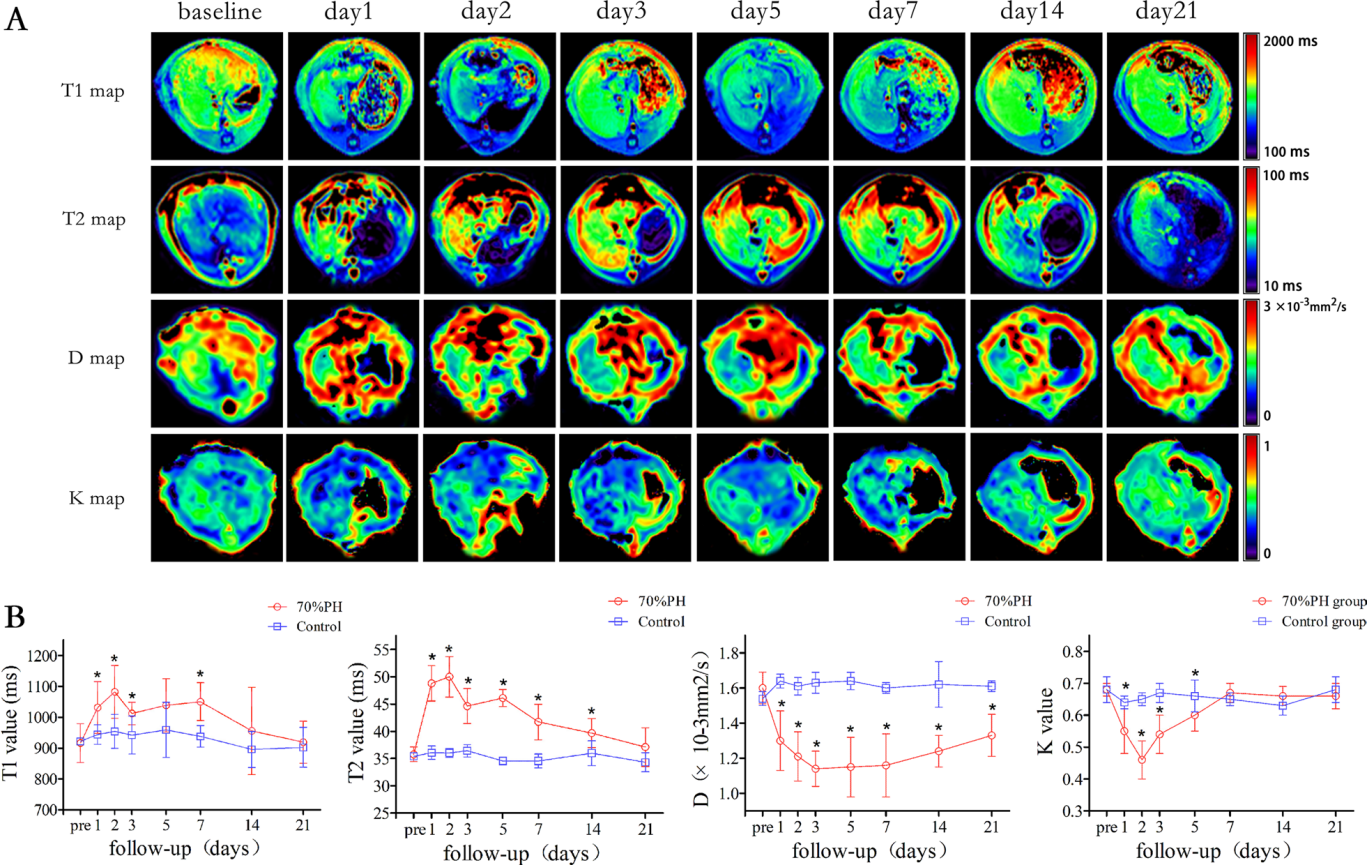
**A**, representative cropped T1, T2, D, and K parametric maps of the regenerating liver from preoperative and postoperative rats; **B**, Changes of T1,T2,D and K values after PH. Error bars represent the standard deviations of the mean values. *Significant changes compared with control group

Representative parametric mapping of T1, T2, D, and K for one rat with PH are shown in Fig. 2A. The means and standard deviations for the T1, T2, D, and K changes and comparisons across different time points are listed in Table 1. All MR parameters, including T1, T2, D, and K values, showed significant changes immediately after PH and then gradually recovered towards the preoperative baseline with the end of regeneration.

**Table 1:** MR parameters of MRph and MRctrl groups changes and comparison across different time points

Time point	T1 (ms)		T2 (ms)		D (× 10^–3^ mm^2^/s)		K	
	MRph	MRctrl	MRph	MRctrl	MRph	MRctrl	MRph	MRctrl
Baseline	916.0 ± 62.9	921.8 ± 8.4	35.8 ± 1.4	35.4 ± 0.6	1.60 ± 0.09	1.54 ± 0.04	0.68 ± 0.02	0.68 ± 0.04
Day 1	1031.3 ± 84.2	943.7 ± 31.7	48.8 ± 3.2^*#^	36.1 ± 1.2	1.30 ± 0.17^*#^	1.64 ± 0.04	0.55 ± 0.07^*#^	0.65 ± 0.02
Day 2	1081.8 ± 85.2^*^	954.0 ± 54.7	50.00 ± 3.7^*^	36.1 ± 0.8	1.21 ± 0.14^*^	1.61 ± 0.05	0.46 ± 0.06^*^	0.64 ± 0.02
Day 3	1011.74 ± 36.7	941.9 ± 62.1	44.6 ± 3.2^*#^	36.4 ± 1.1	1.14 ± 0.10^*^	1.63 ± 0.06	0.54 ± 0.06^*^	0.65 ± 0.02
Day 5	1038.5 ± 86.1	958.7 ± 89.9	46.1 ± 1.6^*^	34.5 ± 0.6^#^	1.15 ± 0.17^*^	1.64 ± 0.05	0.60 ± 0.05^*^	0.67 ± 0.03
Day 7	1050.1 ± 61.3^*^	937.8 ± 34.6	41.7 ± 3.2^*#^	34.6 ± 1.3	1.16 ± 0.18^*^	1.60 ± 0.03	0.67 ± 0.03	0.66 ± 0.05
Day 14	955.3 ± 141.8^#^	895.3 ± 58.8	39.7 ± 2.7^*^	36.0 ± 2.3^#^	1.24 ± 0.09^*^	1.62 ± 0.13	0.66 ± 0.03	0.65 ± 0.02
Day 21	918.8 ± 68.4	902.1 ± 64.9	37.1 ± 3.6^#^	34.3 ± 1.8^#^	1.33 ± 0.12^*^	1.61 ± 0.03	0.66 ± 0.04	0.63 ± 0.03
F	3.947	1.469	22.587	3.300	8.771	2.213	19.802	2.225
*P*	0.04	0.201	< 0.001	0.006	< 0.001	0.063	< 0.001	0.052

Data are shown as mean and standard deviation. D, corrected apparent diffusion; K, kurtosis

^*^Data are significantly different compared to baseline

^#^Data are significantly different compared to the adjacent time points

Both T1 and T2 values significantly increased post-PH and increased to the maximum on day 2 (both *P* < 0.001 vs. baseline), and then returned towards their baseline values. T1 values recovered to the preoperative level on day 14 (*P* > 0.05 vs. baseline), T2 values recovered to the preoperative level on day 21 (*P* > 0.05). The T2 values were significantly different from the adjacent time points on days 1, 3, 7, and 14 (all *P* < 0.001 vs. adjacent time points). T1 values were significantly higher than the MRctrl group on days 1, 2, 3, and 7 (all *P* < 0.05) (Fig. 2B). T2 values were significantly higher compared to the MRctrl group for all time points except day 21 (all *P* < 0.001) (Fig. 2B).

Both D and K values significantly decreased post-PH, D values decreased to a minimum value on day 3 (*P* < 0.001 vs. baseline), then gradually increased. The K values decreased to a minimum value on day 2 (*P* < 0.001), then gradually increased, and the value increased to the baseline value on day 7 and demonstrated no significant difference compared to baseline by day 7 (*P* > 0.05). D values were significantly higher compared to the MRctrl group for all time points (all *P* < 0.05) (Fig. 2B). K values were considerably higher than the MRctrl group on days 1 ~ 5 (all *P* < 0.05) (Fig. 2B).

Intraclass correlations in the MRph group for T1, T2, D, and K were 0.856, 0.931, 0.948, and 0.851, respectively.

### Liver volume analysis

Post-PH, liver volume recovered rapidly, and increased significantly on postoperative days 1 ~ 5, demonstrating a significant difference compared to the adjacent time points (all *P* < 0.001) (Table 2). The growth rate of liver volume began to slow down on postoperative days 7, and the liver volume demonstrated no significant difference compared to the adjacent time points (*P* > 0.05). On day 21, liver volume was approximately 80% of preoperative liver volume (Table 2).

**Table 2:** Changes and comparison of liver volume and pathologic parameters related to liver regeneration

Time point	LV (cm^3^)	HTS (um)	Ki-67 indices (%)
Baseline	10.23 ± 0.56	197.54 ± 18.04	4.61 ± 1.08
Day 1	4.84 ± 0.42^*#^	537.02 ± 38.69^*#^	31.78 ± 1.16^*#^
Day 2	6.06 ± 0.61^*#^	464.11 ± 28.35^*#^	44.52 ± 2.02^*#^
Day 3	7.01 ± 0.73^*#^	452.6 ± 54.69^*^	19.22 ± 1.39^*#^
Day 5	7.87 ± 0.66^*#^	408.66 ± 17.57^*^	11.38 ± 1.33^*#^
Day 7	8.31 ± 0.56^*^	367.76 ± 25.41^*^	8.51 ± 0.78^*#^
Day 14	8.79 ± 0.33^*^	304.31 ± 25.05^*#^	5.08 ± 1.29^#^
Day 21	9.21 ± 0.65^*^	215.57 ± 19.01	4.35 ± 0.98
F	115.080	93.381	903.031
*P*	< 0.001	< 0.001	< 0.001

Data are shown as mean and standard deviation. LV, Liver volume; HTS, Hepatocyte size

^*^Data are significantly different compared to baseline

^#^Data are significantly different compared to the adjacent time points

### Histopathological analysis

**Fig. 3:**
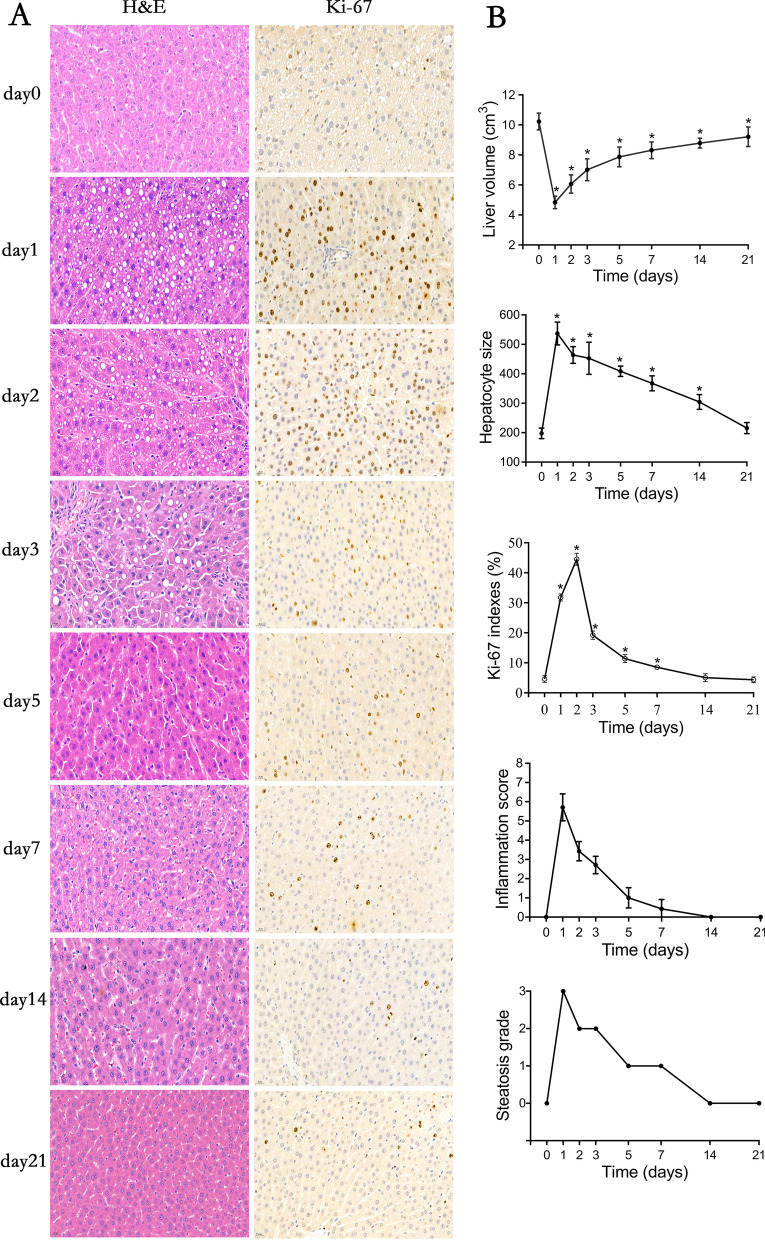
Characteristics of liver tissue preoperative and postoperative. **A**, Hematoxylin–eosin staining (× 400) and Ki-67 proliferation activity staining (× 400). **B**, Liver volume growth, hepatocyte size, Ki-67 proliferation indexes, inflammation score, and steatosis grade. Error bars represent the standard deviations of the mean values. ^*^ Data are significantly different compared to baseline

There were notable morphological alterations over the time course of liver regeneration. The cytoplasm of hepatocytes in the remnant liver was swollen due to fluid and lipid infiltration, indicating hepatocyte hypertrophy after PH. Fat quantification showed that steatosis of liver remnant tissue was most apparenton day 1 (S3 stage), and still evident on days 2 to 3 (S2 stage). It decreased significantly on days 5 to 7 (S1 stage) and recovered to the preoperative level on day 14 (S0 stage) (Fig. 3A). The inflammation of the residual liver was very obvious on day 1 post-resection (5.71 ± 0.70), and then gradually reduced with liver regeneration and tissue repair, and basically returned to normal on day 7 (0.43 ± 0.49) (Fig. 3B). Post-PH, hepatocyte size was significantly larger compared with the baseline on day 1 and reached a maximum (*P* < 0.001), then decreasing to near-baseline by day 21 (*P* > 0.05) (Fig. 3B, Table 2). The representative immunohistochemical images depict Ki-67 positive hepatocytes after PH (Fig. 3A). Ki-67 indices were significantly higher post-PH than baseline, reaching a maximum on day 2 (*P* < 0.001) and returning to near-baseline by day 14. No significant increase in positive hepatocytes was observed in the baseline data (Table 2).

### Correlation analysis

**Fig. 4:**
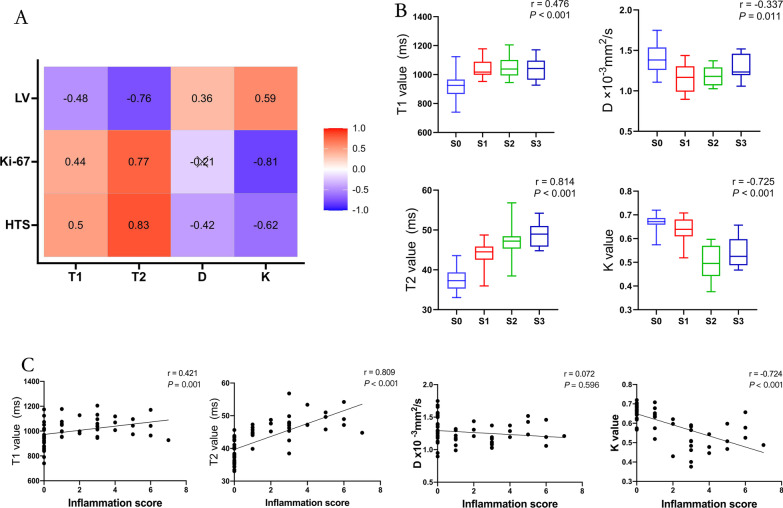
**A**, Correlation heatmaps for quantitative MR imaging parameters and pathological measurements related to liver regeneration. LV, liver volume; HTS, hepatocyte size; **B**, Spearman correlation between magnetic resonance imaging (MRI) parameters and steatosis grade; **C**, Spearman correlation between MR parameters and inflammation score

Both T2 and K values correlated with liver regeneration-related indices (Table 3, Fig. 4). T2 values showed a strong correlation with liver volume (r = − 0.764; *P* < 0.001), steatosis grade (r = 0.814; *P* < 0.001), inflammation (r = 0.809; *P* < 0.001), Ki-67 indices (r = 0.765; *P* < 0.001), and hepatocyte size (r = 0.830; *P* < 0.001). K values had better correlations with Ki-67 indices (r = − 0.807; *P* < 0.001), inflammation (r = − 0.724; *P* < 0.001), and steatosis grade(r = − 0.725; *P* < 0.001) than with liver volumes (r = 0.595; *P* < 0.001), and hepatocyte size (r = − 0.615; *P* < 0.001). A moderate correlation was observed between T1 values and liver volume (r = − 0.481; *P* < 0.001), Ki-67 indexes (r = 0.444; *P* < 0.001), steatosis grade (r = 0.476; *P* < 0.001), inflammation (r = 0.421; *P* = 0.001), and hepatocyte size (r = 0.495; *P* < 0.001). However, D values were only moderately correlated with hepatocyte size (r = − 0.415; *P* < 0.001) and were not or were weakly correlated with other parameters.

**Table 3:** Correlations between MR and pathologic analysis parameters

Characteristic	T1(ms)		T2(ms)		D (× 10^−3^mm^2^/s)		K	
	r	*P* value	r	*P* value	r	*P* value	r	*P* value
LV (cm^3^)	− 0.481	< 0.001	− 0.764	< 0.001	0.364	0.006	0.595	< 0.001
Ki-67 (%)	0.444	< 0.001	0.765	< 0.001	− 0.208	0.124	− 0.807	< 0.001
Hepatocyte size (µm)	0.495	< 0.001	0.830	< 0.001	− 0.415	< 0.001	− 0.615	< 0.001
Steatosis grade	0.476	< 0.001	0.814	< 0.001	− 0.337	0.011	− 0.725	< 0.001
Inflammation	0.421	0.001	0.809	< 0.001	− 0.072	0.596	− 0.724	< 0.001

Significant results are in bold

D, corrected apparent diffusion; K, kurtosis; LV, Liver volume

r = : |0.0–0.2|, very weak to negligible correlation; |0.2–0.4|, weak correlation; |0.4–0.7|, moderate correlation; |0.7–0.9|, strong correlation; and |0.9–1.0|, very strong correlation

## Discussion

This study demonstrated that the multi-parametric MRI monitoring of liver regeneration in the standard rat model is feasible. Moreover, the regeneration process of the residual liver had a good correlation between MRI parameters and histopathological characteristics. These significant relationships have the potential to reveal and elucidate the complex process of residual liver regeneration and have the possible use of these parameters as non-invasive biomarkers to monitor liver regeneration.

Previous studies concluded that liver regeneration after PH is mainly contributed by hepatocyte proliferation [20]. However, Miyaoka et al. [7] found that the volume growth of residual liver after PH was caused by both hypertrophy and proliferation of hepatocytes. Ki-67 immunostaining can mark liver cells in the mitotic stage of liver tissue and has been widely used in animal studies on liver regeneration [7, 16, 17, 21, 22]. In this study, we found that both hepatocyte size and Ki-67 proliferation indices significantly increased after PH, indicating that both processes are involved in liver regeneration, which are consistent with Miyaoka’s studies [7]. The liver volume and hepatocyte size increased significantly on day 1. A large number of Ki-67 positive cells were also observed, suggesting that residual liver tissue could start regeneration quickly to compensate for the loss of liver to maintain normal function. The liver volume continued to increase on day 2, and Ki-67 positive cells also continued to increase and reached the maximum, indicating the regeneration peak, which was consistent with a previous animal study [7, 23]. However, after 70% PH, de Graaf et al. [5] found peak Ki-67 indices on day 1 and Meier et al. [22] found the peak Ki-67 indices on day 3. These inconsistencies may be due to experimental differences, including animal species (rat vs. mice), anesthesia methods, animal ages, and operation times [24]. The third day was a turning point when the regeneration rate decreased, and the number of Ki-67 positive cells started to fall. The liver volume began to increase slowly on day 7, and Ki-67 positive cells also reduced to a shallow level, demonstrating that the liver regeneration reached a plateau on day 7, followed by slow growth until the end of regeneration, which agrees with a study by Hockings et al. [17] These findings may have important implications for future research on the timing of drug intervention in liver regeneration. In addition, interestingly, we found that the corresponding MR parameters also showed a similar trend; T1 and T2 values increased significantly on day 1, reached the maximum on day 2, and began to decline on day 3. K value began to decrease on day 1, came to the minimum on day 2, and increased on day 3. It returned to the preoperative level on day 7, and the subsequent changes were not significant. These results indicate that MR parameters can reflect the changing trend of the liver regeneration process.

In our study, we measured T1 and T2 values of liver parenchyma before and after surgery, and the preoperative baseline values we measured were basically in keeping with a previous study [16]. In addition, we found that T1 and T2 values increased first and then gradually recovered to baseline, which was also in line with the results of Hockings et al. [17]. However, these published studies on rats assessed T1 and T2 values post-PH without detailed data on corresponding pathological results. The increased T1 values reflect the tissue water accumulation due to edema and inflammation after PH [10]. While T2 values are sensitive to an increase in hepatocyte water content and free water fractions [9]. Our histological findings found that significant swelling and steatosis occurred in the first few days after PH and then gradually decreased, based on the observed changes in hepatocyte size throughout the study. Moreover, it has long been known that liver regeneration leads to the rapid accumulation of intracellular lipids in mouse models of PH, suggesting that liver steatosis is an essential liver regeneration process [21]. Cassinotto et al. [25] have shown a correlation between liver T2 values and the degree of liver steatosis, which is in good agreement with our results. In addition, we found that liver T1 and T2 values had a moderate and high correlation with liver inflammation activity separately, which was consistent with the results of Liu et al. in an animal model of liver fibrosis [26]. We speculated that inflammatory reactions such as tissue edema and inflammatory cell infiltration caused by acute liver injury contributed to the increase of T1 and T2 values. Thus, the significant increase in T2 values after PH is due to a combination of residual liver steatosis and increased water content. Increases in fat content significantly decrease T1 values, reducing the ability of the increase in postoperative water content to prolong T1 [27]. This may have interfered with an increase in postoperative T1 values in our study. Moreover, we also found that both T1 and T2 values were correlated with hepatocyte size and Ki-67 indices. Potentially elevated cellular hypertrophy resulting from swollen hepatocytes, a higher number of proliferating, and enhanced lipid content might account for the changed T1 and T2 values in post-PH livers. These results indicate that both T1 and T2 values can be used to monitor the dynamic changes of the liver regeneration process after PH.

Previously, Eberhardt et al. [16] used apparent diffusion coefficients (ADC) derived from conventional diffusion-weighted imaging to evaluate changes in liver parenchyma after PH, and found that the ADC of liver parenchyma decreased after PH. However, ADC is calculated using a monoexponential analysis, which assumes Gaussian behavior of water diffusion. DKI provides a model to quantify non-Gaussian water diffusion. It is more sensitive to the states of liver diffusivities and tissue microstructural complexity compared to standard DWI. The parameter D derived from DKI represents the corrected apparent diffusion. The D values of tissues is affected by cell perimeter length, cell density, size, and permeability, and a study showed that the cell perimeter length is the most important factor [28, 29]. In this work, the D values were significantly decreased at all time points, indicating cellular hypertrophy with limited diffusivity. Moreover, the correlation analysis showed that D value had a moderate negative correlation with hepatocyte size. Thus, the decrease in D may be explained by the increases in cell perimeter length due to cellular hypertrophy. K values are a measure of the complexity and heterogeneity of the cell microenvironment. K values were significantly reduced after PH, in line with the results of Sheng et al. [29] Residual hepatic sinusoids dilate, the arrangement of the sieve plate disappears after PH, exposing the surface of hepatic parenchymal cells to the portal vein, resulting in a relative reduction in tissue complexity [30]. A reduction in tissue complexity may explain our observation that K values decreased in the early postoperative period. However, hepatocellular hypertrophy often affects the microstructure, which may not be explained by a simple Gaussian or restricted diffusion model. Further studies are necessary. In addition, the correlation analysis showed that K value had a good correlation with liver regeneration-related indices (liver volume, steatosis grade, Ki-67 indices, inflammation, and hepatocyte size), indicating the potential value of K values in reflecting liver regeneration processes.

This study had several limitations. First, the kinetics of regeneration differs between humans and rodents, and the results of this study may not fully represent human liver regeneration after PH. Second, imaging animals were not subjected to histological analysis to achieve dynamic and continuous acquisition of MR parameter data. Third, this study analyzed liver regeneration after hepatectomy in normal liver, but liver regeneration in the context of other liver conditions is different. Finally, although we used the B1-corrected variable flip angle (VFA) technique to measure T1 values, it is not sufficient to completely eliminate the effect of confounding factors, further studies comparing other techniques are needed.

## Conclusions

This study used multi-parametric MRI, including the T1 and T2 mapping and DKI, to longitudinally observe rat liver regeneration after PH. The MR parameters showed a consistent change trend with pathological indicators, and correlated with liver regeneration-related indices. Multi-parametric MRI can detect microstructural changes in the residual liver after PH. These techniques might provide non-invasive, sensitive, and quantitative tools for the characterization of residual liver regeneration processes.

## Data Availability

The datasets used and analysed during the current study are available from the corresponding author on reasonable request.

## Abbreviations

MRI: Magnetic resonance imaging
PH: Partial hepatectomy
DKI: Diffusion kurtosis imaging
PA: Pathologic analysis
ROI: Regions of interest
HE: Hematoxylin–eosin
HTS: Hepatocyte size
LR: Liver regeneration
ADC: Apparent diffusion coefficients
VFA: Variable flip angle
